# Validation of the Chinese Version of the Self-Objectification Beliefs and Behaviors Scale

**DOI:** 10.3389/fpsyg.2021.724187

**Published:** 2021-09-24

**Authors:** Min Lang, Yiduo Ye

**Affiliations:** School of Psychology, Fujian Normal University, Fuzhou, China

**Keywords:** self-objectification, young Chinese women, measurement, validity, reliability

## Abstract

Given the limitations of the existing tools used for measuring self-objectification in China, this study aims to validate the Chinese version of the self-objectification beliefs and behaviors scale (C-SOBBS). In this study, we first translated and culturally adopted SOBBS to the Chinese context. We conducted two wave surveys. In the first-wave survey, we recruited 331 female college students whose age ranged from 18 to 35 (*M*_age_=20.28, *SD*=2.99) to complete an online survey that included demographic questions, C-SOBBS, and four other scales to assess the validity of C-SOBBS. In the second-wave survey, 76 participants who took part in the first-wave survey completed the C-SOBBS at a two-week interval for the assessment of test-retest stability. A confirmatory factor analysis was performed to validate the factor structure of the C-SOBBS. The relationship between the C-SOBBS, its factors, and four other measures demonstrated that the C-SOBBS has a convergent and discriminant validity. Furthermore, the results of hierarchical multiple regression demonstrated the C-SOBBS’s incremental validity related to the Female Questionnaire of Trait Self-Objectification and Objectified Body Consciousness-Surveillance subscale. Additionally, the internal consistency and test-retest reliability of the C-SOBBS were also verified. The results of this study demonstrate the utility of the C-SOBBS in assessing the self-objectification beliefs and behaviors of young Chinese women within the context of Chinese culture.

## Introduction

Bartky (1990) proposed the concept of sexual objectification and defined it as “the separation of one’s body, body parts, and sexual functions from one’s identity, thereby reducing a person to the status of an object.” Fredrickson and Roberts (1997) argued that western culture is full of sexual objectification, such as close-ups of women’s sexual body parts in various forms of visual media and men treating women as sexual objects even in interpersonal situations. Women exposed to this kind of influence for a prolonged period will gradually accept and internalize these objectifying attitudes and view their own body from a third person’s perspective, which leads to self-objectification. According to objectification theory, women practicing self-objectification habitually monitor their own physical appearance (Fredrickson and Roberts, 1997), which leads to body image issues, such as body shame (Fredrickson and Roberts, 1997; Adams et al., 2017; Schaefer et al., 2018; Baildon et al., 2021), body dissatisfaction (Grippo and Hill, 2008; Schaefer et al., 2018), physical anxiety (Fredrickson and Roberts, 1997; Jongenelis et al., 2014; Adams et al., 2017), disordered eating (Cohen et al., 2018; Schaefer and Thompson, 2018; Kilpela et al., 2019), depression (Jones and Griffiths, 2015; Register et al., 2015; Vencill et al., 2015), and sexual dysfunction (Fredrickson and Roberts, 1997; Tiggemann, 2011). Physical shame as a common consequence of self-objectification has been extensively studied. For example, Choma et al. (2009) found that self-objectification can predict body shame among Canadian undergraduate women. Similarly, the predictive effect of self-objectification on body shame has also been observed among young Chinese women (Sun and Zheng, 2016; Teng et al., 2019; Wang et al., 2020).

In the two decades after Fredrickson and Roberts (1997) proposed the construct of self-objectification, five self-report scales were devised to assess it, namely, the Self-Objectification Questionnaire (SOQ; Fredrickson et al., 1998), the Body Surveillance Subscale of the Objectified Body Consciousness Scale (OBC-Surveillance; McKinley and Hyde, 1996), the Self-Objectification Scale (SOS; Talmon and Ginzburg, 2016), the Self-Objectification Beliefs and Behaviors Scale (SOBBS; Lindner and Tantleff-Dunn, 2017), and the Female Questionnaire of Trait Self-Objectification (FQSO; Wu and Lang, 2019).

In the SOQ, self-objectification is defined as valuing observable physical appearance (e.g., “How do I look?”) over non-observable physical competence (e.g., “What am I capable of?”). The SOQ items include 10 body attributes, including five appearance-based attributes (e.g., weight) and five competence-based attributes (e.g., health; Fredrickson et al., 1998). Participants were asked to rank the 10 body attributes from 0=*least impact on my physical self-concept* to 9=*great impact on my physical self-concept*. To obtain a final score, the sum of the ranks assigned to the five competence-based attribute scores is subtracted from the sum of the ranks given to appearance-based scores (Fredrickson et al., 1998). A score greater than 0 indicates a greater emphasis on physical appearance, while a score less than 0 indicates a greater emphasis on physical competence (Fredrickson et al., 1998). Although the SOQ is widely used, there are still some shortcomings, mainly in the following aspects. First, the item ranking method forces participants to put physical competence in direct opposition to physical appearance (Hill and Fischer, 2008; Calogero, 2011); however, as a stable trait, at least two other situations may also exist: valuing both physical competence and physical appearance, and valuing neither physical competence nor physical appearance (Wu and Lang, 2019). Second, the item rating method makes it difficult for participants to complete the SOQ correctly; therefore, many questionnaires are unsuitable for data analysis (Calogero, 2011; Lindner and Tantleff-Dunn, 2017; Wu and Lang, 2019). Third, the SOQ’s internal consistency coefficient cannot be calculated because of its rank-order format and scoring system (Hill and Fischer, 2008).

The OBC-Surveillance is a subscale of the Objectified Body Consciousness Scale (OBCS) developed by McKinley and Hyde in 1996. The OBC-Surveillance is mainly used to measure the degree and extent to which a woman perceives herself as an external observer would (McKinley and Hyde, 1996). It includes eight items (e.g., “I think more about how my body feels than how my body looks”). According to objectification theory, self-objectification may cause self-surveillance, which then leads to negative attitudes toward oneself, such as body dissatisfaction and body shame (Fredrickson and Roberts, 1997). Although self-objectification and self-surveillance are similar in concept, different scholars have different views on whether they are interchangeable or equivalent (Calogero, 2011; Lindner and Tantleff-Dunn, 2017). Tiggemann and Kuring (2004) used the SOQ score to predict the OBC-Surveillance score, surmising that self-surveillance is an expected outcome of self-objectification. Some argue that body surveillance is equivalent to self-objectification, so they used OBC-Surveillance alone to measure the latter (Augustus-Horvath and Tylka, 2009; Lindner et al., 2012). In short, it is not clear whether self-surveillance can fully reflect self-objectification (Lindner and Tantleff-Dunn, 2017). The definition of self-objectification includes not only the third-party perspective of the observer but also the excessive value placed on physical appearance over physical competence, as well as treating the body as if it is the only thing that represents the self (Lindner and Tantleff-Dunn, 2017). Thus, OBC-Surveillance is inadequate and inappropriate for measuring self-objectification.

The SOS was developed to assess the nullifying experience of self-objectification by Talmon and Ginzburg (2016). In the SOS, self-objectification is defined as “a state in which individuals perceive themselves as objects and instruments to satisfy the needs and desires of others” (Talmon and Ginzburg, 2016). It includes two factors: invisibility and lack of autonomy, and 17 items (e.g., “Many times people ignore my feelings”). The total score was obtained by averaging all items. Higher scores indicated higher self-objectification (Talmon and Ginzburg, 2016). Unlike the SOQ and the OBC-Surveillance, which assess self-objectification as representing self-perception based on sexual and bodily appearance, the SOS assesses self-objectification as reflecting dehumanization (Talmon and Ginzburg, 2016). Therefore, the SOS cannot be used to measure self-objectification, which focuses on physical appearance.

The SOBBS is a 14-item measure with a 5-point response format. It comprises two factors: the observer’s perspective (“thinking the body as an observer would”) and the body as self (“treating the body as if it is capable of representing the self as a person”; Lindner and Tantleff-Dunn, 2017, p. 256). The total score was obtained by averaging the item scores. Higher scores indicated higher self-objectification (Lindner and Tantleff-Dunn, 2017). The SOBBS has several advantages. The first and most important one is that the conceptual definition of self-objectification is more complete (Lindner and Tantleff-Dunn, 2017). Lindner and Tantleff-Dunn (2017) integrated conceptual and operational definitions of self-objectification and sexual objectification into a single measure. Therefore, the SOBBS can measure both the internalized observer’s perspective (as was done in OBC-Surveillance), the value of physical appearance over physical competence (as was done in the SOQ), and treating the body as if it is the sole representation of the self (as is highlighted by the definition of sexual objectification). Second, each item of the SOBBS is a statement; this form is better understood than the body attributes used by the SOQ (Wu and Lang, 2019). Third, Lindner and Tantleff-Dunn (2017) found that the SOBBS can better predict physical shame and appearance anxiety than the SOQ and OBC-Surveillance.

Based on the SOQ, Wu and Lang (2019) designed the FQSO specifically to measure self-objectification in Chinese women. It includes 17 items, 10 about body-appearance attributes (e.g., facial features and facial shape) and 7 body-competence attributes (e.g., body flexibility). Unlike the SOQ, the FQSO uses a 7-point Likert scale for scoring. To some extent, it overcomes some shortcomings of the SOQ, including its inability to calculate internal consistency reliability and a high rate of incomplete questionnaires. However, there are still some shortcomings. For example, some participants thought the test was unnecessary because they believed that all body attributes were important (Wu and Lang, 2019). Some believed that certain items (e.g., health) were important to all people, and some participants found it confusing to use words as items. Furthermore, when evaluating self-objectification, the FQSO still adopts the definition of “valuing physical appearance more than physical competence,” which is similar to the SOQ. This definition may not fully summarize the complexity of self-objectification; therefore, underlying structural problems still exist in the questionnaire.

Overall, all five measurements were used to assess self-objectification and reported satisfactory reliability and validity (McKinley and Hyde, 1996; Fredrickson et al., 1998; Talmon and Ginzburg, 2016; Lindner and Tantleff-Dunn, 2017; Wu and Lang, 2019). Although the SOQ and the OBC-Surveillance are the most commonly used scales, they are also the most criticized ones (Calogero, 2011; Wu and Lang, 2019). The SOS is not suitable for measuring self-objectification concerning appearance (Talmon and Ginzburg, 2016). The FQSO, although developed specifically for measuring self-objectification in Chinese women, cannot fully measure all the connotations of self-objectification, focusing on appearance (Wu and Lang, 2019). The SOBBS is the most suitable tool for fully assessing self-objectification, which focuses on appearance. However, there is no Chinese version of the SOBBS. Hence, this study intends to adopt and validate the SOBBS to suit the Chinese context.

### The Present Study

This study aims to establish a Chinese version of the SOBBS (C-SOBBS) and to verify the psychometric properties of the translated scale. First, we translate and culturally adopt SOBBS for the Chinese context. Second, we conduct a confirmatory factor analysis (CFA) to evaluate the factor structure of the C-SOBBS. We then evaluate the scale’s convergent and discriminant validity through its relations with the FQSO, body surveillance, body shame, and sexual objectification. Next, we evaluate the incremental validity of the C-SOBBS relative to the FQSO and OBC-Surveillance. Finally, we establish the test-retest reliability of the C-SOBBS using a smaller sample of 76 adult Chinese women. The specific hypotheses for this study are as follows: (1) The C-SOBBS would demonstrate the best fit through CFA for a sample of young Chinese women. (2) The “observer’s perspective” factor would be moderately correlated with the “body as self” factor, and both would be highly correlated with the C-SOBBS total score. (3) The C-SOBBS and its factors are positively correlated with the FQSO and OBC-Surveillance. (4) The C-SOBBS and its factors are positively correlated with body shame and sexual objectification. (5) The C-SOBBS predicts body shame above the FQSO and OBC-Surveillance. (6) The first-wave survey (T1) of the C-SOBBS and its factors would be highly correlated with the second-wave survey (T2) of the C-SOBBS and its factors.

## Materials and Methods

### Translation and Cultural Adaptation of the SOBBS to the Chinese Context

According to the guidelines for cross-cultural adaptation of instruments (Beaton et al., 2000; Swami and Barron, 2019), we first obtained the original English version of the SOBBS and then contacted three professional translators to translate it. They first translated SOBBS independently; after the translation, they held a discussion to get a Chinese version of the SOBBS that all of them agreed on. We then asked two other translators to translate the text back into English. After that, we invited two translators and three experts in self-objectification to discuss the semantic, habitual, cultural, and conceptual equivalence between the original and the Chinese version of SOBBS, named C-SOBBS 1. Next, we invited eight people who studied body image and scale development to provide feedback about the equivalence between the original and C-SOBBS 1 on a 7-point scale (1=not at all and 7=completely equivalent). If the mean of the item was less than 6, we modified it until the mean reached 6 and thus arrived at C-SOBBS 2. Thereafter, we interviewed five university students who were not psychology students and asked them to give feedback on the sentence intelligibility and semantics of 14 items. Finally, we obtained C-SOBBS 3 based on feedback and discussion with two experts.

### Participants and Procedure

Human participation in this study was reviewed and approved by the Fujian Normal University Ethics Committee. For data collection, we adopted convenience sampling and conducted the survey twice. In the first round, we developed an online survey that included demographic questions, C-SOBBS 3, and four other scales through the WJX.cn platform. Then, we contacted some colleagues who taught public courses at universities to help us recruit 339 female college students to complete the online survey between classes. All participants signed an informed consent form before completing the questionnaire. They were also told to complete the survey within 10min. After completing the questionnaire, we gave small gifts as compensation and invited participants who were willing to participate in the retest to provide their contact information. A total of 100 participants left their contact information, and 76 of them participated in the second survey 2weeks later. All data in the second survey were used to analyze the test-retest reliability. In the first round, only 331 correctly completed surveys were used for data analysis because eight surveys were deleted due to the completion time being too short (<5min) and/or incorrect answers to items with specified options. Participants’ age ranged from 18 to 35years (*M*_age_=20.28, *SD*=2.99). The participants were mainly recruited from a university in Zigong City, Sichuan Province and a university in Fuyang City, Anhui Province. The CFA model evaluated in this study has 76 degrees of freedom. A sample size of 331 was sufficient for the analyses based on the sample size guidelines published by MacCallum et al. (1996). The test-retest sample size (*n*=76) in this study was larger than that (*n*=55) in the original SOBBS study. This demonstrates that a sample size of 76 was sufficient.

## Measures

### The Chinese Version of Self-Objectification Beliefs and Behaviors Scale

The C-SOBBS, translated and culturally adapted, was used to measure self-objectification beliefs and behaviors among young Chinese women. The scale consists of 14 items [e.g., “I try to imagine what my body looks like to others (i.e., like I am looking at myself from the outside)”]. Participants rated each of the 14 items on a 5-point scale ranging from 1 (strongly disagree) to 5 (strongly agree). The overall score was obtained by averaging all the item scores. Higher scores indicate stronger self-objectification beliefs and more self-objectification behaviors. The internal consistency of the SOBBS was 0.91 (Lindner and Tantleff-Dunn, 2017). The satisfactory construct validity of the SOBBS was originally reported by Lindner and Tantleff-Dunn (2017).

### The Female Questionnaire of Trait Self-Objectification

The FQSO (Wu and Lang, 2019) was used to measure the degree of trait self-objectification in Chinese women. It includes two factors: physical appearance and physical competence. It has 17 body attributes (e.g., hair, eyes, height, body flexibility, and strength). Participants were asked to rate each item on a 7-point scale, ranging from 1 (least important) to 7 (most important). The overall score was derived by subtracting the competency-based score from the appearance-based score. Higher scores indicated higher trait self-objectification. For Chinese female undergraduate students, Wu and Lang (2019) reported that the internal consistency reliability of the physical appearance factor was 0.89, and the physical competency factor was 0.82. In the current study, Cronbach’s alpha was 0.87 for the factor of physical appearance and 0.89 for the factor of physical competencies. Wu and Lang (2019) reported satisfactory construct validity for a sample of young Chinese women.

### The Body Surveillance Subscale (OBC-Surveillance)

The OBC-Surveillance assesses the degree to which women assume an observer’s perspective of their bodies. It comprises eight items (e.g., “I often worry about whether the clothes I am wearing make me look good”). Respondents rated each of the eight items on a 7-point response scale ranging from 1 (strongly disagree) to 7 (strongly agree). The total score was obtained by summing the item scores. Higher scores indicated a stronger tendency to take an outsider’s perspective while viewing their own body. For Chinese undergraduate students, the internal consistency reliability of the OBC-Surveillance was 0.88 (Chen and Jiang, 2007). For American young women and middle-aged women, the internal consistency reliability of the OBC-Surveillance was 0.89 (McKinley and Hyde, 1996). In this study, the internal consistency of the OBC-Surveillance was 0.81. McKinley and Hyde (1996) originally reported the construct validity of the OBC, and the satisfactory construct validity of the Chinese version of the OBC-Surveillance scale was also demonstrated by Chen and Jiang (2007).

### The Body Shame Subscale

The BSS is a subscale of the OBCS (McKinley and Hyde, 1996). It assesses the degree to which women feel that they are bad people when they view themselves as not meeting cultural appearance standards, particularly thinness. It comprises eight items (e.g., “When I can’t control my weight, I feel like something must be wrong with me”). Respondents rated each of the eight items on a 7-point response scale ranging from 1 (strongly disagree) to 7 (strongly agree), as in McKinley and Hyde (1996). The total score was obtained by summing the item scores. Higher scores indicated higher body shame. For Chinese undergraduate students, the internal consistency reliability of the OBC-BSS was 0.86 (Chen and Jiang, 2007). Schaefer et al. (2018) reported similar reliability estimates (white college women: α=0.80; black college women: α=0.75). In the current study, the internal consistency of the BSS was 0.82, and McKinley and Hyde (1996) originally reported the construct validity of the OBC, and satisfactory construct validity of the Chinese version of the BSS was also demonstrated by Chen and Jiang (2007).

### The Interpersonal Sexual Objectification Scale

The ISOS is used to measure the frequency of sexual objectification experienced within the past year (Kozee et al., 2007). It comprises 15 items (e.g., “How often have you noticed someone staring at your breasts when you were talking to them?”) with a 5-point response format (ranging from 1=never to 5=always). The total score was obtained by summing the item scores. Higher scores indicated greater experience of sexual objectification. For Chinese undergraduate students, the internal consistency reliability of the ISOS was 0.87 (Sun and Zheng, 2016). In the current study, Cronbach’s alpha is 0.91, and satisfactory construct validity was originally demonstrated by Kozee et al. (2007). Sun and Zheng (2016) also reported satisfactory construct validity in the Chinese version.

### Data Analyses

We examined the factor structure of the C-SOBBS using CFA performed in AMOS 22.0. To evaluate the model fit, widely accepted fit indices were used as: the goodness-of-fit index (GFI; near or above 0.90), comparative fit index (CFI; near or above 0.95), the ratio of the chi-square to the degree of freedom (χ^2^/df), and the root mean square error of approximation (RMSEA; at or below 0.06) with a 90% confidence interval (CI; Hu and Bentler, 1999). The validity was performed in three ways: (a) Pearson’s correlations were used among the two factors of C-SOBBS, overall C-SOBBS, and four other scales, (b) a set of estimations of composite reliability (CR) and average variance extracted (AVE) of each factor were examined, and (c) a hierarchical multiple regression was conducted to analyze the incremental validity of the C-SOBBS relative to the FQSO and OBC-Surveillance. We assessed internal consistency reliability with Cronbach’s alpha and test-retest reliability with the intraclass correlation coefficient (ICC) using the commonly reported cutoff values of 0.70 and 0.80 (Nunnally, 1978; Fleiss, 1999).

## Results

### Descriptive and Correlation Statistics

As presented in Table 1, the mean for 14 items of C-SOBBS ranged from 2.05 to 3.23, and the standard deviation (SD) for 14 items of C-SOBBS ranged from 0.81 to 1.04. For both dimensions, all items had values of kurtosis (ranges from −0.79 to 0.75) and skewness (ranges from −0.40 to 0.88), indicative of slight deviations from the normal distribution. According to the literature review by Kline (1998), there are no sensitivity problems or significant non-normality. There were significant correlations among 14 items of C-SOBBS which ranged from 0.23 to 0.58 (*p*<0.01).

**Table 1:** Descriptive statistics and correlations among items.

	Item1	Item2	Item3	Item4	Item5	Item6	Item7	Item8	Item9	Item10	Item11	Item12	Item13	Item14
Mean	2.98	2.37	3.23	2.26	3.03	2.05	2.87	3.08	2.96	2.07	2.35	2.05	2.70	2.05
*SD*	0.96	0.91	0.92	0.86	0.97	0.81	1.04	1.01	0.97	0.89	0.96	0.93	1.02	0.96
Kurtosis	−0.27	0.05	−0.07	0.61	−0.57	0.24	−0.79	−0.54	−0.56	0.23	0.02	0.75	−0.64	0.41
Skewness	−0.12	0.48	−0.40	0.65	−0.12	0.60	−0.10	−0.40	−0.08	0.68	0.51	0.88	−0.10	0.83
Item1	–													
Item2	0.42^**^	–												
Item3	0.41^**^	0.42^**^	–											
Item4	0.29^**^	0.53^**^	0.32^**^	–										
Item5	0.36^**^	0.35^**^	0.47^**^	0.37^**^	–									
Item6	0.28^**^	0.42^**^	0.27^**^	0.55^**^	0.31^**^	–								
Item7	0.40^**^	0.36^**^	0.49^**^	0.34^**^	0.47^**^	0.35^**^	–							
Item8	0.48^**^	0.42^**^	0.53^**^	0.37^**^	0.54^**^	0.38^**^	0.58^**^	–						
Item9	0.37^**^	0.38^**^	0.42^**^	0.38^**^	0.44^**^	0.31^**^	0.38^**^	0.50^**^	–					
Item10	0.28^**^	0.36^**^	0.24^**^	0.48^**^	0.29^**^	0.46^**^	0.30^**^	0.34^**^	0.35^**^	–				
Item11	0.32^**^	0.31^**^	0.23^**^	0.37^**^	0.31^**^	0.33^**^	0.27^**^	0.32^**^	0.33^**^	0.45^**^	–			
Item12	0.29^**^	0.40^**^	0.30^**^	0.43^**^	0.28^**^	0.54^**^	0.35^**^	0.35^**^	0.30^**^	0.52^**^	0.39^**^	–		
Item13	0.45^**^	0.41^**^	0.48^**^	0.38^**^	0.46^**^	0.45^**^	0.51^**^	0.58^**^	0.43^**^	0.42^**^	0.43^**^	0.58^**^	–	
Item14	0.26^**^	0.35^**^	0.24^**^	0.45^**^	0.24^**^	0.42^**^	0.33^**^	0.30^**^	0.27^**^	0.47^**^	0.41^**^	0.49^**^	0.43^**^	–

*SD, Standard deviation*;

** *p<0.01.*

### Validation of the C-SOBBS’s Factor Structure

A CFA with maximum likelihood estimation was conducted to confirm the two-factor structure of the 14-item C-SOBBS. The model provided an excellent fit for the sample, GFI=0.93, CFI=0.95, RMSEA=0.06 [90% CI (0.048, 0.073)], and χ^2^/df was statistically significant for the model, χ^2^/df=2.19, *p*<0.001. To prove that the two-factor model would be better than the single-factor model, we also conducted a CFA to confirm the one-factor structure of the 14-item C-SOBBS, GFI=0.84, CFI=0.86, RMSEA=0.10. These results indicate that the two-factor C-SOBBS demonstrated the best fit for the young Chinese women sample, thereby supporting Hypothesis 1. The standardized regression weights of each item on the corresponding latent factor were all significant and valued between 0.56 and 0.79 (Table 2).

**Table 2:** Item standardized regression weights, squared multiple correlations (SMC), and item descriptive statistics for the C-SOBBS (*n*=331).

Item	Weight	SMC
Observer’s perspective		
1. I have thoughts about how my body looks to others even when I am alone	0.59	0.35
3. I try to imagine what my body looks like to others (i.e., like I am looking at myself from the outside)	0.67	0.44
5. I choose specific clothing or accessories based on how they make my body appear to others	0.66	0.43
7. When I look in the mirror, I notice areas of my appearance that I think others will view critically	0.70	0.49
8. I consider how my body will look to others in the clothing I am wearing	0.79	0.63
9. I often think about how my body must look to others	0.62	0.38
13. I try to anticipate others’ reactions to my physical appearance	0.75	0.56
Body as self		
2. Looking attractive to others is more important to me than being happy with who I am inside	0.62	0.39
4. How I look is more important to me than how I think or feel	0.71	0.51
6. My physical appearance is more important than my personality	0.70	0.49
10. My physical appearance says more about who I am than my intellect	0.68	0.47
11. How sexually attractive others find me says something about who I am as a person	0.56	0.32
12. My physical appearance is more important than my physical abilities	0.71	0.50
14. My body is what gives me value to other people	0.64	0.41

### Validity

As presented in Table 3, both the “observer’s perspective” factor and the “body as self” factor had significant positive correlations with overall C-SOBBS (*r*=0.92 and *r*=0.89, *p*<0.01), and the correlation between the factors was 0.64 (*p*<0.01). There was a significant moderate correlation between the two factors and a significantly high correlation between each factor and the overall C-SOBBS. Further, the correlation between the two factors was lower than the correlation between the two factors and the overall C-SOBBS, which supports Hypothesis 2. The composite reliability (CR) of two factors of C-SOBBS was 0.86 and 0.84, and the AVE was 0.47 and 0.44, respectively. The data show that acceptable reliability indices were achieved (Fornell and Larcker, 1981), and the minimum AVE (0.44) was greater than the square of the correlation coefficient of the two factors (0.41). The results show that the C-SOBBS has high convergent and discriminant validity. Furthermore, the two factors and the overall C-SOBBS had significant positive correlations with the FQSO and the OBC-Surveillance, supporting Hypothesis 3. This supports the convergent validity of C-SOBBS. In addition, the “observer’s perspective” factor, the “body as self” factor, and the overall C-SOBBS had significant positive correlations with BSS and ISOS, supporting Hypothesis 4, indicating that the C-SOBBS has good criterion validity. In this study, we also conducted hierarchical multiple regression to analyze whether the C-SOBBS predicted body shame beyond the two measures used to assess self-objectification in China—the FQSO and OBC-Surveillance. As presented in Table 4, the FQSO predicted the body shame in Model 1. In model 2, both FQSO and OBC-Surveillance predicted body shame. In model 3, the FQSO and C-SOBBS predicted body shame, whereas the OBC-Surveillance did not. Collectively, the addition of C-SOBBS to the regression model results in a significant improvement in the prediction of body shame. Thus, Hypothesis 5 is validated. Taken together, these results provide strong evidence for the validity of C-SOBBS.

**Table 3:** Correlations among the measures.

Measures	Correlations						
	1	2	3	4	5	6	7
C-SOBBS_F1	–						
C-SOBBS_F2	0.64^**^	–					
C-SOBBS_Total	0.92^**^	0.89^**^	–				
FQSO	0.35^**^	0.50^**^	0.46^**^	–			
OBC-Surveillance	0.62^**^	0.51^**^	0.63^**^	0.49^**^	–		
BSS	0.32^**^	0.45^**^	0.40^**^	0.40^**^	0.38^**^	–	
ISOS	0.20^**^	0.29^**^	0.14^**^	0.14^*^	0.20^**^	0.13^*^	–
*M*	2.98	2.17	2.57	−0.86	3.85	3.43	22.35
*SD*	0.72	0.65	0.62	0.93	0.75	0.90	7.29
Possible scores	1 to 5	1 to 5	1 to 5	−6 to 6	1 to 15	1 to 7	1 to 15

*C-SOBBS_F1, Chinese version of Self-Objectification Beliefs and Behaviors-Observer’s Perspective; C-SOBBS_F2, Chinese version of Self-Objectification Beliefs and Behaviors-Body as Self; C-SOBBS_Total, Chinese version of Self-Objectification Beliefs and Behaviors-Total Score; FQSO, Female Questionnaire of Trait Self-Objectification; OBC-Surveillance, the Self-Surveillance Subscale; BSS, Body Shame Subscale; and ISOS, Interpersonal Sexual Objectification Scale*.

** *p<0.01;*

* *p<0.05*.

**Table 4:** Incremental validity of C-SOBBS relative to the FQSO and OBC-surveillance scale.

Outcome variable	Δ*R*^2^	Δ*R*^2^(*F*)	*B*	*SE B*	*β*	*t*
BSS						
Model 1	0.16	62.37^***^				
FQSO			3.10	0.39	0.40	7.90^***^
Model 2	0.05	18.96^***^				
FQSO			2.17	0.44	0.28	4.96^***^
OBC-Surveillance			0.30	0.07	0.25	4.35^***^
Model 3	0.03	14.40^***^				
FQSO			1.79	0.44	0.23	4.05^***^
OBC-Surveillance			0.14	0.08	0.12	1.83
C-SOBBS			2.80	0.74	0.24	3.80^***^

*C-SOBBS, Chinese version of Self-Objectification Beliefs and Behaviors-Total Score; FQSO, the Female Questionnaire of Trait Self-Objectification; OBC-Surveillance, Objectified Body Consciousness Scale Self-Surveillance Subscale; BSS, Objectified Body Consciousness Scale Body Shame Subscale; and ISOS, Interpersonal Sexual Objectification Scale*.

*** *p<0.001*.

### Reliability

The final 14-item C-SOBBS shows excellent internal consistency. Cronbach’s alpha was 0.86 for the “observer’s perspective” factor and 0.84 for the “body as self” factor, and the overall internal consistency of C-SOBBS was 0.90. To establish the test-retest reliability of the C-SOBBS over a two-week interval, 76 participants completed the survey. The results of the ICC (3.1) indicate that the test-retest reliability of the overall C-SOBBS (0.86), the observer’s perspective factor (0.74), and the factor of the “body as self” (0.72) were excellent. Thus, Hypothesis 6 is validated.

### General Discussion

The primary goal of our study was to translate and culturally adapt the SOBBS to the Chinese context, specifically for young Chinese women, to address some limitations of the existing measures of self-objectification. To achieve this, we performed several steps. First, we translated the SOBBS into Chinese and obtained the Chinese version of the SOBBS. Unlike the SOQ (Fredrickson et al., 1998) and the FQSO (Wu and Lang, 2019) that use body attributes as items, the C-SOBBS utilizes unambiguous statements as items. These are more readily understood than body attributes, and they also avoid situations where body attributes do not capture group experiences. The Chinese version offers a new instrument for testing self-objectification in Chinese women to answer calls from Moradi (2010) and Calogero (2011) to clarify the construct and refine the assessment. We validated the two-factor structure of the C-SOBBS *via* CFA and evaluated the scale’s construct and convergent validity, as well as incremental validity relative to the FQSO and OBC-Surveillance. Furthermore, we analyzed the internal consistency reliability and test-retest reliability of the C-SOBBS and its factors. The CFA results support the fit of the replicable two-factor model. Additionally, by analyzing the relationship between the overall C-SOBBS and its factors with other Chinese-language self-report measures of trait self-objectification, namely, the FQSO (Wu and Lang, 2019) and the OBC-Surveillance (McKinley and Hyde, 1996), as well as body shame assessed by the BSS of OBCS (McKinley and Hyde, 1996) and sexual objectification measured by the ISOS (Kozee et al., 2007), the results demonstrate that the factor of observer’s perspective, the factor of the “body as self,” and the overall C-SOBBS were positively correlated with the FQSO, OBC-Surveillance, BSS, and ISOS. In addition, the correlation between the C-SOBBS and its factors is higher than the correlation between the C-SOBBS factor 1 (observer’s perspective) and C-SOBBS factor 2 (body as self). These findings are consistent with the theoretical assumptions and provide evidence regarding the validity of the C-SOBBS as a suitable tool to measure self-objectification in young Chinese women. Additionally, the results of the hierarchical multiple regression demonstrate the incremental validity of the C-SOBBS. Furthermore, the stability of C-SOBBS by comparing two administrations of C-SOBBS over a two-week interval suggests that C-SOBBS scores are stable and indicative of a trait construct.

In general, the study presented in this paper provides evidence of a strong and replicable factor structure, construct, convergent, and incremental validity, as well as test-retest reliability for the Chinese version of the SOBBS.

### Limitations and Future Research Directions

This research has several strengths, including the finding of evidence of reliability and validity for the Chinese version of the SOBBS. Nevertheless, several limitations of this study must be acknowledged.

First, the study relies exclusively on self-reported data, which may be susceptible to social desirability bias (Morgado et al., 2017), even though all the measures we use report high internal reliability. Second, our sample consists of female college students aged 18–35years, with an average age of 20.28. The distribution of different age groups is inconsistent; the age group of 25–35years is significantly under-represented compared to the 18–25 age group. Moreover, we did not recruit adolescent girls, older women, or men to participate in our study. Future studies need to further expand the sample size to verify the structure of C-SOBBS in older female groups as well as in adolescent girls and men.

Furthermore, the establishment of validation for any measure is an ongoing process. Although this study provides evidence of the reliability and validity of C-SOBBS among young Chinese women aged 18–35, it is still unclear whether its factor structure applies to other age groups. Future psychometric evaluations should investigate the issue of discriminant validity.

### Practical Implications

The findings of this study have several important practical implications, including clinical ramifications. A substantial body of the literature shows that self-objectification is directly related to women’s mental health. Women with a high level of self-objectification are associated with a high risk of physical anxiety (Tiggemann and Andrew, 2012; Watson et al., 2012), body dissatisfaction (Lindner et al., 2012; Tiggemann and Andrew, 2012; Brock et al., 2021), body shame (Tiggemann and Boundy, 2008; Choma et al., 2009; Schaefer et al., 2018; Baildon et al., 2021), depression (Peat and Muehlenkamp, 2011; Jones and Griffiths, 2015; Register et al., 2015), disordered eating (Schaefer and Thompson, 2018; Al-Mutawa et al., 2019; Kilpela et al., 2019; Holmes et al., 2020), and sexual dysfunction (Fredrickson and Roberts, 1997; Tiggemann, 2011). Counselors and therapists can use C-SOBBS to help people deal with issues related to self-objectification. Additionally, by focusing on attitudes and behaviors representative of self-objectification, C-SOBBS helps us identify potential intervention targets and can also serve to improve people’s self-awareness of their tendencies to interpret how others may view their bodies while cultivating an appreciation for other positive aspects beyond physical appearance (Tylka and Augustus-Horvath, 2011; Calogero and Tylka, 2014; Lindner and Tantleff-Dunn, 2017).

## Conclusion

In China, parallel to the increased prevalence of eating disorders and medical cosmetology, many researchers have conducted in-depth studies on self-objectification. However, the absence of a reliable and validated measurement tool specifically constructed to assess self-objectification is a serious impediment to accurate and high-quality research. The purpose and primary achievement of this article is to address this gap by validating an additional instrument adapted specifically to measure young Chinese women’s self-objectification. In this study, we provide sufficient reliability and validity for C-SOBBS, concluding that it is an effective tool with the potential to foster further research that will advance related theories, research, and practices.

## Data Availability Statement

The raw data supporting the conclusions of this article will be made available by the authors, without undue reservation.

## Ethics Statement

The studies involving human participants were reviewed and approved by the Fujian Normal University Ethics Committee. The patients/participants provided their written informed consent to participate in this study.

## Author Contributions

ML: conceptualization, formal analysis, and writing. YY: conceptualization, editing, and supervision. All authors contributed to the article and approved the submitted version.

## Conflict of Interest

The authors declare that the research was conducted in the absence of any commercial or financial relationships that could be construed as a potential conflict of interest.

## Publisher’s Note

All claims expressed in this article are solely those of the authors and do not necessarily represent those of their affiliated organizations, or those of the publisher, the editors and the reviewers. Any product that may be evaluated in this article, or claim that may be made by its manufacturer, is not guaranteed or endorsed by the publisher.
